# Reinforcement learning-based control for waste biorefining processes under uncertainty

**DOI:** 10.1038/s44172-024-00183-7

**Published:** 2024-02-29

**Authors:** Ji Gao, Abigael Wahlen, Caleb Ju, Yongsheng Chen, Guanghui Lan, Zhaohui Tong

**Affiliations:** 1 School of Chemical & Biomolecular Engineering, Georgia Institute of Technology, 311 Ferst Dr, Atlanta, 30318 GA USA; 2 H. Milton Stewart School of Industrial and Systems Engineering, Georgia Institute of Technology, 755 Ferst Drive, Atlanta, 30332 GA USA; 3 School of Civil and Environmental Engineering, Georgia Institute of Technology, 790 Atlantic Dr, Atlanta, 30332 GA USA

**Keywords:** Climate change, Environmental impact

## Abstract

Waste biorefining processes face significant challenges related to the variability of feedstocks. The supply and composition of multiple feedstocks in these processes can be uncertain, making it difficult to achieve economically feasible and sustainable waste valorization for large-scale production. Here, we introduce a reinforcement learning-based framework that aims to control these uncertainties and improve the efficiency of the process. The framework is tested on an anaerobic digestion process and is found to perform better than traditional control strategies. In the short term, it achieves faster target tracking with increased precision and accuracy, while in the long term, it shows adaptive and robust behavior even under additional seasonal supply variability, meeting downstream demand with high probability. This reinforcement learning-based framework offers a promising and scalable solution to address uncertainty issues in real-world biorefining processes. If implemented, this framework could contribute to sustainable waste management practices globally, making waste biorefining processes more economically viable and environmentally friendly.

## Abstract (web summary)

Modelling waste biorefining processes can be difficult due to variability of feedstocks, making process optimization challenging. To address these uncertainties, Ji Gao and colleagues introduced a reinforcement learning-based framework, using a model anaerobic digestion process to demonstrate their control methods.

## Introduction

Waste has emerged as a significant challenge to both local and global sustainable development. According to projections, annual solid waste generation is expected to increase by 70% by 2050, reaching 3.4 billion tons per year^1^. Unfortunately, a substantial amount of the waste is not properly managed, resulting in loss of resources and land, greenhouse gas (GHG) emissions, environmental pollution, and adverse health effects. Currently, non-sanitary landfills are the primary waste disposal method, accounting for over 50% of global waste processing^2^. The uncontrolled waste disposal in landfills causes the quality degradation of soil due to leachate penetration, surface runoffs, and particulate matters from harmful chemicals. Landfills also generate 1.6 billion tons of carbon dioxide (CO2) equivalent GHG emissions, making them one of the largest anthropogenic sources of GHG emissions^2^. Waste incineration, another common waste management method, emits a large amount of toxic pollutants, including dioxins and furans, into the atmosphere. To minimize resource loss and mitigate the negative climate and environmental impact, there has been a growing interest in harnessing and effectively utilizing waste.

Biorefining processes offer a sustainable solution for effective waste utilization by converting low-cost biomass such as agricultural and forestry residues, food waste, animal manure, and organic fraction of municipal solid waste, into a range of value-added bioproducts, including biochemicals, biomaterials, and bioenergy^3^. Biorefineries integrate waste valorization and decarbonization concepts, reducing the reliance on fossil fuels and limiting the introduction of additional carbon in the carbon cycle, making them a promising approach to achieving a circular economy by closing the loop on waste management, moving towards a zero-waste manufacturing model^4^.

However, biorefining processes face major challenges associated with uncertainties that arise from highly variable feedstocks, fluctuation, and complex process dynamics^5^. Variations in the composition, quality, availability, and external environmental factors, such as weather conditions and market fluctuations, propagate through the process, resulting in significant uncertainty in production. This can lead to serious consequences such as unstable operations, suboptimal process performance, and safety concerns. Traditional control strategies, such as proportional-integral-derivative (PID) based controllers that are the most widely used controllers in industry, often rely on linearization of system dynamics, estimation of the state of the process, and external knowledge of the system, making them unsuitable for handling a highly non-linear process with large uncertainties associated with the feedstock and operating conditions^6–19^. Advanced control techniques, such as model predictive control^20–22^ are hindered by the difficulty of measuring all the intermediate products associated with the complex reactions, due to the partially observable nature of biorefining process reactions. Therefore, a control algorithm capable of dealing with uncertainties that arise from multiple sources in a highly non-linear biorefinery process is needed to increase production efficiency while maintaining stability to ensure optimal production, leading to a resilient market supply for energy, fuel, and other valuable products from renewable, low-cost sources like biomass and waste.

Recent developments in reinforcement learning (RL)^23^ technologies offer new possibilities for dealing with the uncertainties and complex dynamics of biorefinery processes, particularly for centralized biorefineries that use various waste feedstocks. RL has already demonstrated tremendous success in fields like robotics^24^, biomedical sciences^25^, and plasma physics^26^. RL is a model-free approach to decision-making that avoids explicitly estimating the complex dynamical model required by state estimation or prediction-based controllers. This relieves the strict assumptions on models and uncertainties associated with the aforementioned algorithms. RL is an end-to-end decision-making approach, which makes it simpler to analyze and implement than methods that separately estimate the model and select the optimal decision. In addition, the adaptive feature of RL enables the control policy to change adaptively in response to environmental changes or model uncertainties. Several studies have already employed RL in biorefinery processes to control specific uncertainty^27–31^, but these studies fall short in considering control of processes where multiple uncertainties arise simultaneously from multiple feed inputs, distinct feed composition, supply variability, and feed inventory that mirrors actual production situations.

**Fig. 1:**
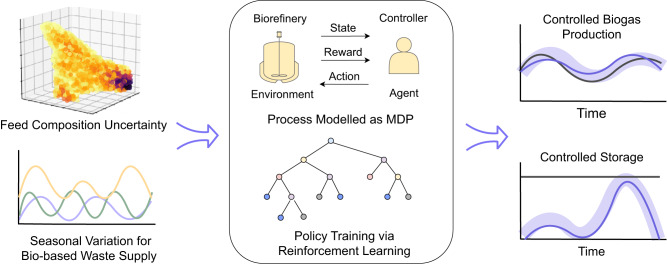
Overview of the data-driven framework for the anaerobic digestion process to control biogas production. The framework is designed for the control of biogas production and storage against the uncertainty associated with composition distribution from various waste feedstocks and the long-term seasonal variation in feed supply. The entire process will be modeled as a Markov decision process (MDP), and subsequently controlled via RL policies. Two control policies are devised for the control of two scenarios, short-term and long-term, that cover the majority of situations during production.

In this study, we developed a integrated framework to address the challenge of uncertainty control in biorefining processes. The framework utilizes data-driven techniques, specifically RL, to achieve stable production despite the uncertainties from various sources both internally and externally (Fig. 1). The framework focuses on two common production situations: 1) short-term variable target tracking, and 2) long-term robust control of fixed production targets with combined inventory control. The framework allows accurate tracking of defined targets with minimal lagging despite the complex reaction and process dynamics. Further, the uncertainties in feedstock availability, storage, and compositions can be handled simultaneously, achieving a robust production with respect to the market demand, and satisfying a customized objective function comprising production targets and costs.

The proposed data-driven reinforcement learning-based framework aims to exploit the value of every data point for a more effective design and integration of the control system. The framework transforms the feedstock uncertainties and complex biorefining processes into probabilistic models to achieve accurate and robust control of biogas production under uncertainties, with reduced effort in conducting lab analysis and understanding the specific reaction mechanism. The effectiveness of our framework was demonstrated using a widely adopted, yet complex biorefining technology, anaerobic digestion (AD) as a case study. This study contributes to the enhancement and stability of more sustainable refinery techniques and allows for more robust production from waste materials.

## Results

### Quantifying risks by uncertainty modeling

**Fig. 2:**
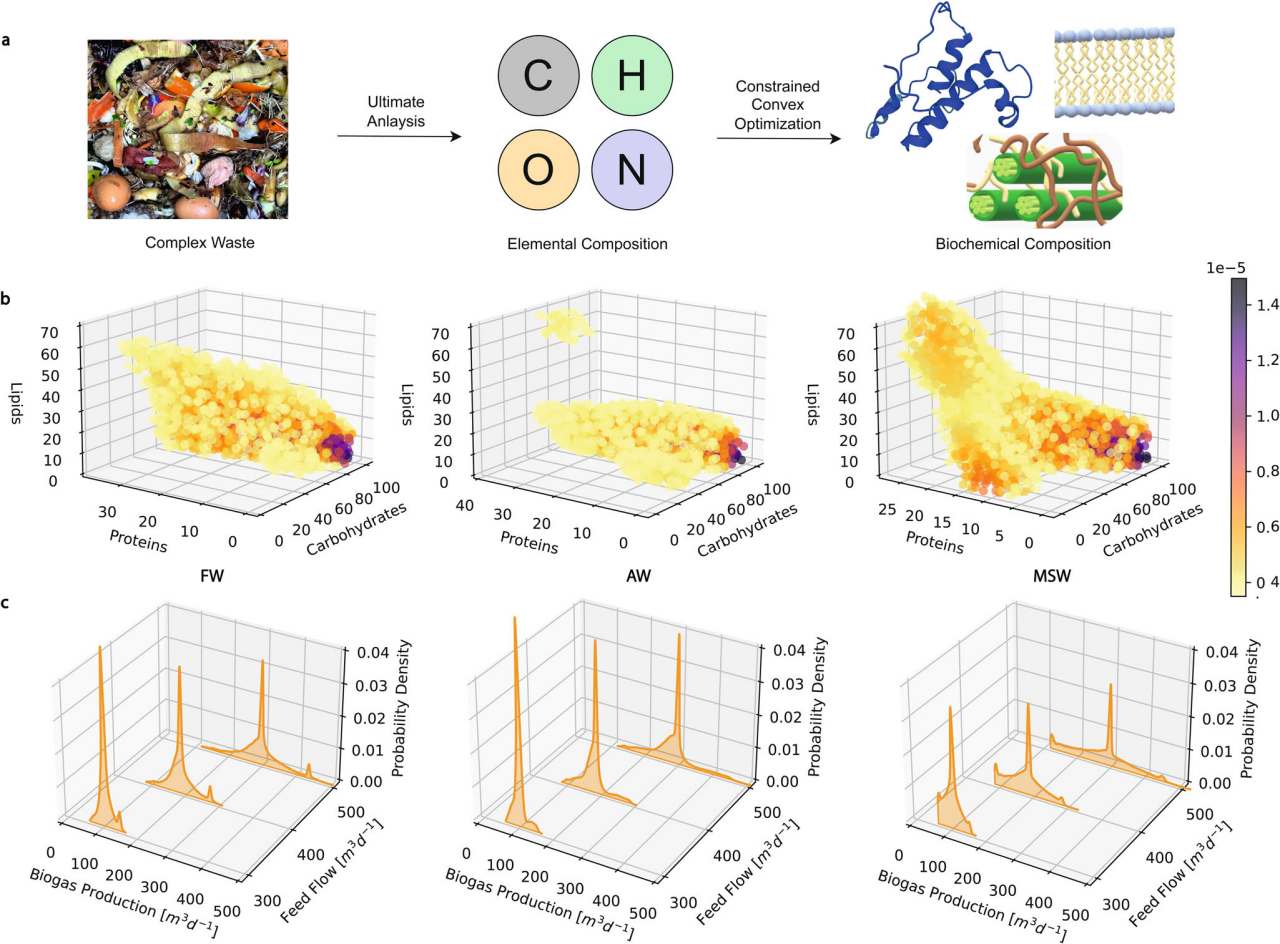
Analysis of feedstock composition distribution. **a** The flowchart shows the process from collecting the ultimate analysis data of different kinds of waste from literature to optimization and estimation of the biochemical compositions (proteins, lipids, and carbohydrates). **b** Resampled distribution of proteins, lipids, and carbohydrates from kernel density estimation model for food waste (FW), agricultural waste (AW), and municipal solid waste (MSW), with units in mass fraction. The probability density at a specific location is indicated by the color of the sample. **c** Probability density distribution of biogas production rate at three different feed flow rates using FW, AW, and MSW as feedstock respectively. Each run was carried out in semi-batch mode with feed addition every 1 day lasting 30 days. The simulation was carried out 10^5^ times.

To evaluate the impact of feedstock uncertainty on biogas production, we used elemental data to characterize FW, AW, and MSW and identified the distribution of the key biochemical components such as carbohydrates, proteins, lipids, and lignin. The use of the elemental compositions of feedstocks does not require complicated multi-step procedures found in a direct biochemical composition analysis. First, a total of 268 data points of elemental compositions were gathered from the literature, with details outlined in Supplementary Data and Supplementary Note 6. We then made the assumption, which is considered reasonable, that within each type of waste, carbon (C), hydrogen (H), oxygen (O), and nitrogen (N) contribute to carbohydrates, proteins, lipids, and lignin (Supplementary Note 5). An optimization problem (Section ''Feed Composition Analysis'') was formulated and solved for each data point with respect to the elemental balances to estimate the mass fractions of the key biochemical components that best fit the ultimate analysis data (Fig. 2a). Each data point is assigned a weight based on its category, following the statistics of waste streams studied by EPA^32^. Finally, we employed the weighted kernel density estimation (KDE) with Gaussian kernels^33^ to derive the distributions of the key chemical components including carbohydrates, lipids, proteins, and lignin in FW, AW, and MSW.

The Anaerobic Digestion Model No. 1 (ADM1) is used for the simulation of reactor dynamics^34^. ADM1 is a widely used mechanistic model for anaerobic digestion processes applied in the field of environmental engineering and wastewater treatment^35^. It describes the biological processes occurring in the digesters, which are used to treat organic waste and produce biogas. ADM1 considers a network of microbial reactions, that can be summarized into four stages, i.e., hydrolysis, acidogenesis, acetogenesis, and methanogenesis, to simulate the conversion of organic matter into biogas^34^. The model assumes the reaction to be carried out in a continuous stirred tank reactor with ideal mixing and accounts for various parameters such as temperature, pH, and inhibition. More details on ADM1 is shown in Supplementary Note 3.

The results in Fig. 2b showed the differences in composition distribution for the three main biogas production waste sources including carbohydrates, proteins, and lipids, with a negative correlation between carbohydrates and proteins and between carbohydrates and lipids in feedstocks. The correlations of different biochemical compositions were visualized by projecting the sampled distribution on a two-dimensional plane (Supplementary Fig. 3).

We found that FW, AW, and MSW have varying biogas potentials due to the different distribution of carbohydrates, proteins, and lipids, which leads to different pathways during the digestion process (Supplementary Note 2 and Supplementary Fig. 1). To quantify this effect, we performed sensitivity analysis by re-sampling the calculated distributions and inputting the samples into the AD model at three different feed flowrates without control. The aggregated results of mean biogas production are shown in Fig. 2c. The distribution of biogas production for all three sources of waste displays some bi-modal behavior, with the existence of a second peak for FW and MSW. FW potentially has the highest biogas production rate due to its high lipid concentration. We employed the coefficient of variation (CoV) as a metric to identify the degree of dispersion of the data. CoV is calculated as the ratio between the standard deviation and the mean of the samples. The large values of the CoV for all three feed sources (FW: 16%, AW: 20%, MW: 22%) indicate a significant spread of the biogas production, emphasizing the necessity of efficient control algorithms for the AD process under variable feedstocks. This effort in uncertainty modeling serves as an important first step for devising the control policy of the AD process.

### Anaerobic digestion in a reinforcement learning setting

To design an effective control policy for a complex AD system, it is essential to have an understanding of both the feed and process characteristics. In Section ''Quantifying risks by uncertainty modeling'', the feedstock distribution analysis and uncertainty modeling enable us to understand the input to the AD process. However, the AD process itself also presents several computational challenges for optimal control, including high process uncertainties, non-linear process dynamics, and low process state observability. To address these challenges, we leverage the Markov property, which assumes that the next state of the system does not depend on the past history when given the present state. By formulating the problem as a Markov decision process (MDP), we can use a data-driven probabilistic model instead of a mechanistic model, which reduces the need for detailed modeling and parameter estimation. This approach allows us to develop efficient numerical methods for solving the problem.

A finite-time MDP of horizon *T* is defined by the tuple $(S,A,{\{{P}_{t}\}}_{t = 1}^{T},{\{{c}_{t}\}}_{t = 1}^{T})$, where *S* is the set of states a system can occupy at any time, *A* is the set of actions to the decision maker, *P*_*t*_: *S* × *A* → Δ_*S*_ is the transition dynamics (where Δ_*S*_ is the probability simplex over *S*) at time *t*, and ${c}_{t}:S\times A\to {\mathbb{R}}$ is the cost/penalty incurred at time *t* for a chosen action at state *s* ∈ *S*. The goal of the decision maker is to find a policy *π*(⋅∣*s*) ∈ Δ_*S*_ for all states *s*, which assigns a probability for selecting an action at a state *s*, that minimizes the cumulative penalty over the entire horizon, where the expectation is taken with respect to the randomness from the transition dynamics and policy (Equ. (1)).

1 $$\mathop{\min }\nolimits_{\pi }J(\pi ):= {\mathbb{E}}\left[\mathop{\sum }\limits_{t=1}^{T}{c}_{t}({s}_{t},{a}_{t})\left\vert \right.{s}_{t+1} \sim {P}_{t}(\cdot | {s}_{t},{a}_{t}),{a}_{t} \sim \pi (\cdot | {s}_{t})\right]$$

Within the context of AD, one can choose the states as $S\subseteq {{\mathbb{R}}}_{+}^{33}$ to model the concentration profiles of all reactants within the AD reactor, the actions as $A\subseteq {{\mathbb{R}}}_{+}^{3}$ for the three controlled feedstock flowrates, the transition dynamics as *P*_*t*_ ≡ *P*(⋅∣*s*_*t*_, *a*_*t*_) defined by the AD process (with feed inputs drawn from an approximate distribution over the uncertain feedstock estimated from Section ''Quantifying risks by uncertainty modeling''), and the costs as ${c}_{t}\equiv {c}_{t}({s}_{t},{a}_{t}):= \parallel \Pi ({s}_{t})-\Pi ({s}_{t}^{* }){\parallel }_{1}$, which is proportional to the MAE, where Π selects a subset of target profiles, e.g., biogas, and ${s}_{t}^{* }$ is target state.

An added complication arises from the fact that not all concentration profiles of the AD process can be observed, which implies that certain states of the MDP cannot be directly observed. To solve this issue, one can utilize a partially observable MDP (POMDP). At a high level, a POMDP incorporates a set of beliefs, or probabilities, over the possible states of the system that the decision-maker perceives. However, since there are over twenty species that cannot be immediately observed in the reactor, even with discretization, the state space and belief space become too large. To overcome this challenge, we identify and exploit critical features as an estimate for the unobservable state variables. This enables us to leverage the MDP formulation without directly estimating all states. Thus, we can employ RL algorithms, which are designed to efficiently solve MDPs, to devise control policies for both short (Section ''Variable production control'') and long-term (Section ''Robust production control'') AD scenarios.

### Variable production control

**Fig. 3:**
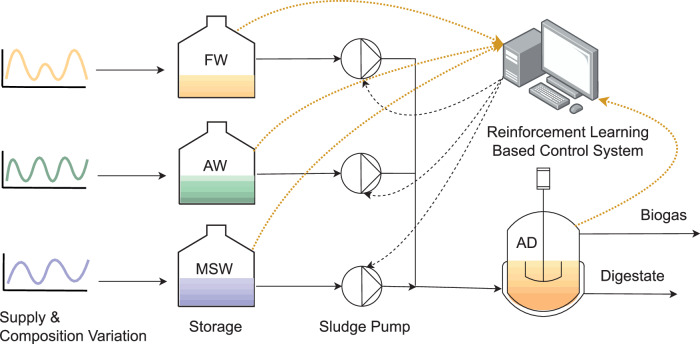
Flow diagram of the simulated centralized biogas production process. The AD process of interest comprises four categories of units: an AD reactor, three reservoirs for incoming waste feedstocks of food waste (FW), agricultural waste (AW), and municipal solid waste (MSW), an RL-enabled control system, as well as electromechanical devices such as sensors, pumps, and actuators. Starting from the upstream supply stored in the waste reservoir, the three feedstocks under uncertainty will be mixed, with their proportion and total volumetric flow rate determined by the control system and fed into the AD reactor. The process will undergo cycles of continuous feeding followed by batch reaction with a semi-batch operation procedure.

We first demonstrated the performance of our framework in controlling the biogas in a centralized AD process (Fig. 3) over a short time period, where target changes are predefined without the effect of long-term supply variation. This can happen in circumstances when precise tracking of the production target is needed, e.g., during the process ramping up stage, the addition or elimination of a feed source, or time periods for demand side management where production is adjusted responding to fluctuations in energy market^36^.

Based on the importance and ease of measurement, the biogas production rate is used as the observation space for the MDP, reliving the necessity of additional sensors for other intermediates in the reactor. We then formulate the state space of the problem by combining two consecutive observations of the biogas production rate. Using consecutive observation inherently incorporates the gradient information into the state, which allows a better estimation of the actual state of the reactor, at the cost of more computational effort due to the enlarged state space. The input flow rates of FW, AW, and MSW are defined as actions. The detailed simulation settings can be found from Supplementary Note 4. A backward dynamic programming (DP) algorithm, trained offline, is used for this finite-horizon control problem. For comparison, we carry out additional experiments with a discrete PID controller.

**Fig. 4:**
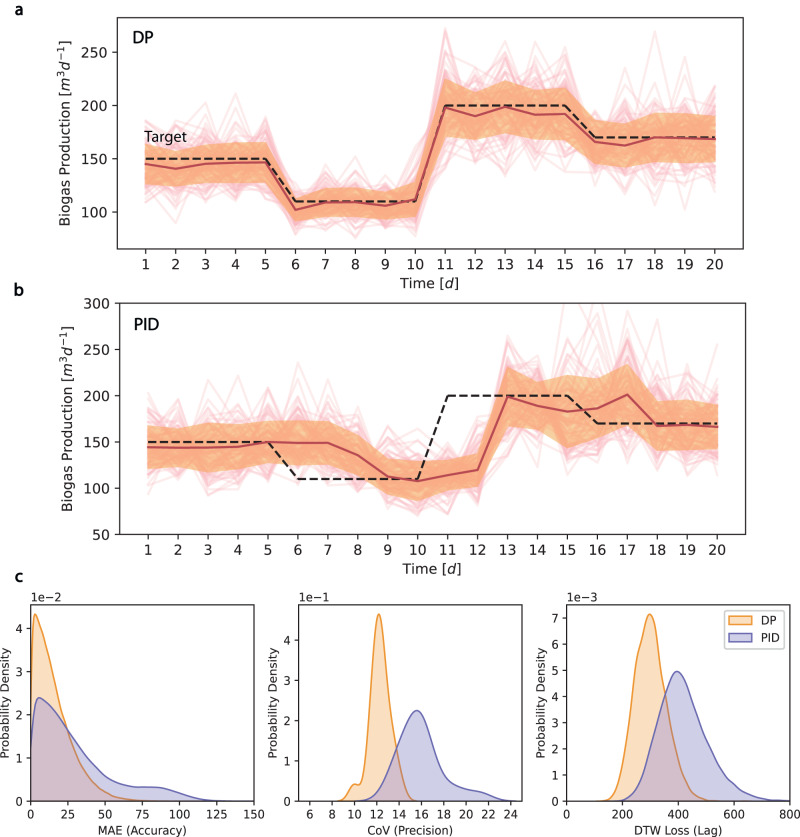
Results and performance metrics of variable biogas production controlled by dynamic programming. **a** Biogas production controlled by dynamic programming (DP) policy. **b** Biogas production controlled by proportional-integral-derivative (PID) policy. **a**, **b** Feed composition variation is considered in this scenario, without seasonal variation. Controlled biogas production for 10^4^ simulations. Black dotted line: production target, red line: mean of the biogas production, orange shaded area: one standard deviation from the mean, pink lines: 100 sample trajectories of simulation results. **c** Performance metrics to quantify accuracy using mean absolute error (MAE), precision using coefficient of variation (CoV) and lag using dynamic time warping loss (DTW) for DP and PID policies. The shaded areas indicate the probability density distribution of errors regarding the performance metrics.

We designed the performance metrics by comparing the actual biogas production with the target production over a predefined time period. Accuracy describes if the desired biogas production target is achieved by calculating the MAE between the actual production and the target. Precision measures the variance or spread of biogas production by calculating the CoV of all simulation runs at each time step. Lag identifies the degree of lagging between the actual production and the target using dynamic time warping (DTW) loss^37,38^ (Supplementary Note 1). To test the framework’s ability to handle uncertainty, we sampled three feed inputs each from a distribution and carried out 10^4^ simulations. Our results show that the RL-based control policy (Fig. 4a), dynamic programming (DP), outperforms a PID policy (Fig. 4b) in all three performance metrics. Specifically, the RL-based policy achieved a 48% increase in accuracy, a 22% increase in precision, and a 28% reduction in lag on average. The distribution of all three metrics from simulation results (Fig. 4c) favored smaller values with narrower and taller peaks, indicating better and more uniform performance. The DP algorithm, which takes advantage of data-driven off-line training, achieved better performance in all three metrics, making it suitable for tracking predefined biogas production targets for a short period of time.

### Robust production control

**Fig. 5:**
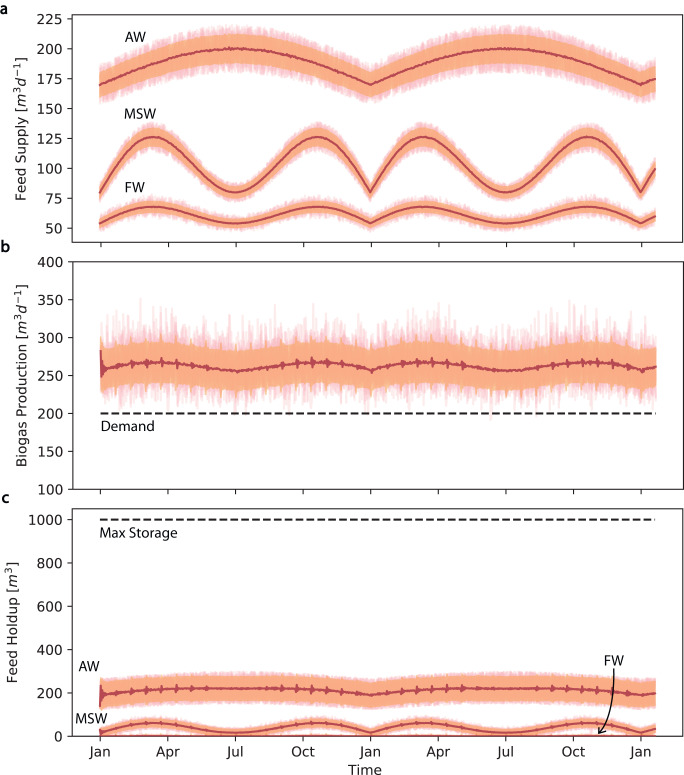
Results for the control of biogas production and inventory with feed seasonal variability by PMD algorithm. **a**–**c** Feed composition variation, seasonal variation, and storage are considered for this scenario. Red line: mean of the simulation results for 10^3^ simulations, orange shaded area: one standard deviation from the mean, pink lines: 10 sample trajectories of simulation results. **a** Simulated seasonal variability of the feed supply for food waste (FW), agricultural waste (AW), and municipal solid waste (MSW), subject to long-term variation with uniform random noise. **b** Biogas production rate. The black dotted line indicates the simulated downstream demand for biogas. **c** Feed holdup in storage tank for AW. The black dotted line indicates the max allowed holdup. Enlarged figures are shown in Supplementary Fig. 2

To improve the resilience of biogas production over a longer period, a policy mirror descent (PMD)^39^ based RL algorithm is applied. PMD is a general class of policy gradient methods to offer state-of-the-art total sample complexity for RL problems by separately handling the bias and expected error of the gradient, which allows one to decrease the bias more quickly. A smaller bias leads to more accurate solutions when the gradient is unknown, as is the case in non-linear dynamic systems such as the AD process. In addition to the feed mixing problem stated in Section ''Variable production control'', we also consider the inventory control problem for separate storage tanks of FW, AW, and MSW under the disturbances in feed supply. This simulates the situation of a centralized co-digestive facility, where the uncertainties come from the feed composition, and also the upstream supply due to seasonal variation (Fig. 5a), with details shown in Supplementary Note 4. Considering the routine production of biogas within the AD facility over a long period of time, we expect to maintain a stable and sufficient biogas production rate at a relatively steady state, subject to the downstream consumer demand. We formulated a cost function (Equ. (2)) with four terms representing the performance of the RL algorithms. The first term applies a mild penalty in mean absolute error (MAE) if the biogas production (*y*^+^) is higher than the defined target (y^*^). The second terms applies a stronger penalty in MSE if the biogas production (*y*^−^) is not meeting the required target, which increases the biogas production robustness. The third term represents the operating cost which is assumed to be proportional to the process throughput denoted by the total flowrate variable (*F*_*T*_). The fourth term represents cost due to storage overflow. It applies a penalty for the wasted feed Δ*x* when the max storage volume of the tank is exceeded.

2 $${{{{{{{\rm{cost}}}}}}}}={w}_{0}({y}^{+}-{y}^{* })+{w}_{1}\parallel {y}^{* }-{y}^{-}{\parallel }_{2}^{2}+{w}_{2}{F}_{T}+{w}_{3}\Delta x.$$

On average, the PMD-based control scheme achieves a demand satisfaction rate of more than 99%, with a mean biogas production rate generally more than one standard deviation above the target (Fig. 5b) and a reduced CoV of 10%. At the same time, the storage for the feed is maintained at a level where overflow seldom happens. Figure 5c shows the storage holdup for FW, AW, and MSW. The storage overflow for all three tanks is almost 0 on average. This finding demonstrates that the policy is robust and can minimize the probability of satisfying demand under large feed uncertainties while reducing the chance of storage overflow.

## Discussion

### Connection with real-world applications

Our framework can be applied to real-world co-digestion facilities with various waste feedstocks. The employment of the RL-based framework in real-world practice requires two crucial sets of information. First, we should know the local waste feedstock composition distribution and their seasonal trends of supply. Second, we need to have the estimation of parameters for the ADM1 model.

The local waste composition distribution plays a crucial role in training the AD process and would change for different regions. Our framework provides an effective approach to estimating this feedstock distribution through an analysis of the historical waste feedstock data specific to the region. To streamline this calculation, we employ the optimization and KDE techniques described in Sections ''Feed composition analysis'' and ''Distribution analysis''. In situations where data is insufficient, we resort to a baseline feedstock model outlined in Section ''Quantifying risks by uncertainty modeling'', which is derived from literature data and distribution patterns. In this context, we utilize a weighted KDE approach, assigning greater weights to local real-world data and lesser weights to the baseline literature model.

The subsequent step to employ our framework in real-world practice involves parameter estimation for the ADM1 model. It is noteworthy that RL can be trained purely using operational data (state, action, disturbance, and action-value pairs) without the need for a mechanistic model. Fitting the ADM1 model for training can be seen as a method for augmenting data with physics-informed insights when a substantial amount of data is unavailable. While ADM1 has demonstrated its effectiveness across various processes, it may overlook certain crucial factors like phosphate, sulfur, heavy metals, microbial activity, and feedstock density^35^. When applying ADM1 to a specific process or region, if these additional complexities are found to be significant, one should consider using ADM1 with modifications to address these factors before parameter estimation. In cases where no such observations are found, direct parameter estimation can be employed. Model modification with parameter estimation proves to be effective across numerous processes^35^. For the practical application of ADM1, historical digestion data can be used to estimate the model parameters in real-time practice.

During the execution stage, we integrate RL using the ADM1 model, feedstock composition distribution, and feedstock seasonal trend as a simulator. The optimal policy resulting from training can subsequently be applied to real-world production processes to enhance stability under various uncertainties such as feedstock compositions and seasonal supply trends. The biogas production rates at predefined time intervals will be recorded as observations. These observations will be fed into the RL optimal policy function, which generates recommendations for actions that can be executed.

### Analysis and future perspectives

We have introduced a data-driven framework for achieving the control of biorefining processes with multiple uncertainties to enhance production resilience. Using a centralized anaerobic co-digestion process as an example, we have developed a framework consisting both short-term and long-term control strategies to address several common situations that could arise during real-world biorefining or waste valorization processes. In this section, we present the analysis and future perspectives of our data-driven framework.

With recent technological advancements in data science, we aim to investigate the use of data for controlling sustainable chemical processes with high uncertainty. In biorefinery processes, especially those utilizing various sources of waste for feed supply, knowledge of the biochemical compositions of waste feedstocks is crucial for the process control and optimization, as the process dynamics are closely related to the reactant components^34^. For the anaerobic digestion process, the carbohydrate, lipid, and protein components in the waste feedstocks determine the process dynamics and biogas generation rate. However, this information is rarely available in literature and waste is often characterized only in the form of elemental analysis, H, C, O, and N compositions^40^. Therefore, we propose a solution that uses optimization techniques to establish the relationship between the biochemical and elemental compositions, followed by machine learning techniques to identify the probabilistic model of the feed biochemical compositions. This data-centric method allows for the estimation and distribution of the complex feedstocks without time-consuming lab analysis in a real-world waste refining and valorization process. This analysis is crucial for designing process control schemes under feedstock variability and for advancing biorefineries under these conditions. Without extensive system controls, waste utilization and biorefining, which have high feed variability and system uncertainty^41^, cannot compete with non-renewable resource production, which has low feed variability and high process stability^42^.

To resolve this issue, we employed data-driven RL algorithms to improve the process resilience in a model-free manner, rather than traditional control schemes such as PID or expert systems that rely heavily on the accuracy of the model and knowledge of the process. Specifically, DP and PMD are applied for the control of the AD process. The potential of these algorithms is not limited to the cases considered in this study, although the existence of a process model is assumed for simulation purposes. For an existing real-world process, the historical operational data such as feed supply, feed composition, reactor sensor signals and production rate can be effectively used for the construction of a transition model and the state-action-value function of the RL algorithms for training or pre-training. This creates an alternative pathway for understanding the complex physical reaction process with a probabilistic model, handling complex interactions and lags associated with process variables such as the concentration profiles within the reaction system.

Moreover, our framework utilizes a policy gradient algorithm, PMD, which employs an online (or model-free) and on-policy scheme with a theoretical justification for its efficiency^39^. This enables adaptive control due to unforeseen changes in process dynamics, operations, reactor environment, or objective functions (costs, production, environmental impacts etc.) for biorefining processes. As mentioned in Section ''Robust production control'', the RL algorithm is usually applied to a process with a fixed target and mechanics. However, the online and on-policy scheme allows the control policy to adaptively change with respect to the variability such as updates in long-term biogas production targets and changes in seasonal temperature, further increasing the resilience of the process. We demonstrated a case study of changing objective function in Supplementary Fig. 4, showing that the new target can be met in a relatively fast manner without re-training the algorithm. The adaptive behavior is controllable by adding a regularization term into the objective function, allowing for regularization of the deviation from the initial control policy at a cost of tuning effort.

Furthermore, the framework has the potential to increase the applicability of a limited data case. Recent advancements in transfer learning and robust learning bring new possibilities for the efficient reuse of the trained models and algorithms in limited data conditions. We have used KDE on the literature data and EPA statistics in the U.S. for feed composition study, formulating a generic or baseline model. The true distribution of feedstock composition differs depending on geolocation, seasons, and feedstock sources. However, we can still utilize the baseline model to reduce the amount of data needed for training at another biogas plant by applying weightings to the samples. Similarly for RL, various transfer learning techniques with modification or mapping of policy, action-value function, and reward^43,44^ can be carried out to reduce the training effort on other AD processes. Applying robust learning techniques^45,46^ can increase the resilience of the RL policies in new environments. The potential of transfer learning and robust learning largely increase the applicability of this framework in real-world processes.

## Method

### Feed composition analysis

We have collected initial data to build a probability distribution that accurately represents the heterogeneity of the feedstock data. In particular, we have focused on the ultimate analysis data, which includes elemental composition (C, H, O, and N) sourced from different categories such as municipal solid waste (e.g., sanitary textiles, office papers), food waste (e.g., coffee grounds, apple peels), and agricultural waste (e.g., rice husk, straw, manure). By utilizing this preliminary data, we were able to determine the corresponding biochemical compositions (protein, lipid, carbohydrate, and lignin) from the data. To estimate the biochemical composition quickly and accurately, we formulated an optimization problem using mass balance relationships.

Within each waste type, we carried out the mass balance calculations on the primary elements C, H, O, and N, as shown in Equ. (3), we obtained the composition of total C, H, O, and N from the ultimate analysis of literature values. The variable *x*_*i*_ represents the composition of element *i* (i.e., sugar, lipids, proteins, and lignin) within the waste. To ensure that the composition of all substances is greater or equal to zero, we added additional bounds.

3 $$	{x}_{{{{{{\rm{sugar}}}}}}}{{{{{{\rm{C}}}}}}}_{{{{{{\rm{sugar}}}}}}}+{x}_{{{{{{\rm{lipids}}}}}}}{{{{{{\rm{C}}}}}}}_{{{{{{\rm{lipids}}}}}}}+{x}_{{{{{{\rm{proteins}}}}}}}{{{{{{\rm{C}}}}}}}_{{{{{{\rm{proteins}}}}}}}+{x}_{{{{{{\rm{lignin}}}}}}}{{{{{{\rm{C}}}}}}}_{{{{{{\rm{lignin}}}}}}}={{{{{{\rm{C}}}}}}}_{{{{{{\rm{total}}}}}}}\\ 	 {x}_{{{{{{\rm{sugar}}}}}}}{{{{{{\rm{H}}}}}}}_{{{{{{\rm{sugar}}}}}}}+{x}_{{{{{{\rm{lipids}}}}}}}{{{{{{\rm{H}}}}}}}_{{{{{{\rm{lipids}}}}}}}+{x}_{{{{{{\rm{proteins}}}}}}}{{{{{{\rm{H}}}}}}}_{{{{{{\rm{proteins}}}}}}}+{x}_{{{{{{\rm{lignin}}}}}}}{{{{{{\rm{H}}}}}}}_{{{{{{\rm{lignin}}}}}}}={{{{{{\rm{H}}}}}}}_{{{{{{\rm{total}}}}}}}\\ 	 {x}_{{{{{{\rm{sugar}}}}}}}{{{{{{\rm{O}}}}}}}_{{{{{{\rm{sugar}}}}}}}+{x}_{{{{{{\rm{lipids}}}}}}}{{{{{{\rm{O}}}}}}}_{{{{{{\rm{lipids}}}}}}}+{x}_{{{{{{\rm{proteins}}}}}}}{{{{{{\rm{O}}}}}}}_{{{{{{\rm{proteins}}}}}}}+{x}_{{{{{{\rm{lignin}}}}}}}{{{{{{\rm{O}}}}}}}_{{{{{{\rm{lignin}}}}}}}={{{{{{\rm{O}}}}}}}_{{{{{{\rm{total}}}}}}}\\ 	 {x}_{{{{{{\rm{sugar}}}}}}}{{{{{{\rm{N}}}}}}}_{{{{{{\rm{sugar}}}}}}}+{x}_{{{{{{\rm{lipids}}}}}}}{{{{{{\rm{N}}}}}}}_{{{{{{\rm{lipids}}}}}}}+{x}_{{{{{{\rm{proteins}}}}}}}{{{{{{\rm{N}}}}}}}_{{{{{{\rm{proteins}}}}}}}+{x}_{{{{{{\rm{lignin}}}}}}}{{{{{{\rm{N}}}}}}}_{{{{{{\rm{lignin}}}}}}}={{{{{{\rm{N}}}}}}}_{{{{{{\rm{total}}}}}}}\\ 	 {x}_{{{{{{\rm{sugar}}}}}}}+{x}_{{{{{{\rm{lipids}}}}}}}+{x}_{{{{{{\rm{proteins}}}}}}}+{x}_{{{{{{\rm{lignin}}}}}}}=1\\ 	 {x}_{{{{{{\rm{sugar}}}}}}},{x}_{{{{{{\rm{lipids}}}}}}},{x}_{{{{{{\rm{proteins}}}}}}},{x}_{{{{{{\rm{lignin}}}}}}}\ge 0$$

The linear system has four variables, five equality constraints, and four non-negativity constraints on variables, making it impossible to solve directly (Equ. (4)). Therefore, we reformulated the problem into a constrained least-square problem, with the objective of minimizing the residual.

4 $$\mathop{\min }\limits_{x}\frac{1}{2}\parallel Ax-b{\parallel }_{2}^{2}\\ s.t.\quad \mathop{\sum}\limits_{i}{x}_{i}=1\\ {x}_{i}\ge 0\\ \forall i\in \{{{{{{{{\rm{sugar}}}}}}}},{{{{{{{\rm{lipids}}}}}}}},{{{{{{{\rm{proteins}}}}}}}},{{{{{{{\rm{lignin}}}}}}}}\}$$

### Distribution analysis

We employed kernel density estimation (KDE) with Gaussian kernel to identify the probability distribution of proteins, lipids, and sugar concentration for each type of waste (i.e., MSW, FW, AW). By using KDE for data analysis, we were able to create a model that better mirrors the distribution of raw data without the need to assume independent and identically distributed variables for uni-variate distribution fitting.

### A reinforcement learning approach to solve the MDP

The goal is to obtain a policy $\hat{\pi }$ that approximately solves problem (1), i.e., $J({\hat{\pi}}) - \mathop{\min}\limits_{\pi}J(\pi) \leq \epsilon$ for some accuracy tolerance *ϵ* > 0. One approach is to use backward induction (or backward dynamic programming). We successively solve for the following value function from *t* = *T* down to *t* = 1, and ${u}_{T+1}^{* }\equiv 0$ is the placeholder terminal reward (Equ. (5)).

5 $${u}_{t}^{* }({s}_{t})=\mathop{\max }\limits_{a\in A}\left\{{r}_{t}({s}_{t},a)+{{\mathbb{E}}}_{{s}_{t+1}}[{u}_{t+1}^{* }({s}_{t+1})]\right\}$$

If the state and action spaces are not too large and the expectation can be computed exactly, such as in a finite support of uncertainty with known probabilities, the problem can be solved efficiently and exactly. However, when controlling the AD process, the true distribution of the transition dynamics may not be known, making RL techniques necessary to solve an MDP. In this study, we use an optimization-based approach that combines PMD with a conditional temporal difference learning method^39,47^. These methods offer start-of-the-art total sampling complexity and ensure the gradient estimation error bias is small, resulting in a more efficient algorithm.

The PMD algorithm is conceptually simple. Starting with an arbitrary initial policy *π*_0_ and iteration counter *k* = 0, PMD finds the next policy *π*_*k*+1_(⋅∣*s*) that solves the minimization problem (Equ. (6)).

6 $$\mathop{\min }\limits_{p(\cdot | s)\in {\Delta }_{| {{{{{{{\mathcal{A}}}}}}}}| }}\left\{{\eta }_{k}[\langle {Q}_{{\pi }_{k}}(s,\cdot ),p(\cdot | s)\rangle +\mu {h}^{p}(s)+{\tau }_{k}{D}_{{\pi }_{0}}^{p}(s)]+{D}_{{\pi }_{k}}^{p}(s)\right\}\,\forall s\in S$$

Then we increment *k* and repeat the update. Here, *Q*_*π*_(*s*, *a*) is called the Q-function that is defined as the expected cost when we start the system at state *s* ∈ *S* with action *a* ∈ *A* and continue running under policy *π*. Since the Q-function is not known in advance, it can be estimated either by Monte Carlo simulation via independent trajectories or a stochastic iterative method such as temporal difference (TD) learning^48^. In our simulation, we utilized a recently developed conditional TD learning that extends TD learning by reducing the bias quickly^47^. The function *h*^*p*^(*s*) is a general convex regularization term with respect to a policy *p* at state *s* that can model constraints or enforce some sparsity in the policy. The distance measures ${D}_{\pi }^{p}$ is the Bregman divergence between two policies *p* and *π*, and the constants {*η*_*k*_, *τ*_*k*_, *μ*} correspond to the step size, perturbation term, and strong convexity term, respectively. The incorporation of a perturbation term helps accelerate the convergence of the algorithm. Since the minimization problem (Equ. (6)) is strongly convex, there exists a unique solution, and it is known in closed form when the Bregman divergence is properly chosen. One such distance is the Kullback-Leibler divergence, or KL-divergence, defined in Equ. (7).

7 $${D}_{{\pi }^{{\prime} }}^{\pi }(s)=\mathop{\sum}\limits_{a\in A}\pi (a| s)\log \frac{\pi (a| s)}{{\pi }^{{\prime} }(a| s)}$$

Suppose the initial policy *π*_0_ is chosen as uniformly random over all actions, i.e., *π*_0_(*a*∣*s*) = 1/∣*A*∣ for all *a* ∈ *A* and *s* ∈ *S*. Then choosing the KL-divergence as the Bregman divergence ${D}_{\pi }^{p}$ and the convex regularization term *h*^*p*^ as zero, the next policy *π*_*k*+1_ can be analytically computed from Equ. (8).

8 $${\pi }_{k+1}(a| s)\propto \exp \left\{\frac{\log ({\pi }_{k})-{\eta }_{k}{Q}_{{\pi }_{k}}(s,a)}{{\eta }_{k}{\tau }_{k}+1}\right\}$$

where the notation ∝ means “proportional to” so that *π*_*k*+1_(⋅∣*s*) is a probability distribution over the actions *A*. Intuitively, the update above implies that if the Q-function at state *s* and action *a* has a large positive value, then the likelihood of selecting action *a* at state *s* in the new policy *π*_*k*+1_ should be decreased. Conversely, if ${Q}_{{\pi }_{k}}(s,a)$ has a small or a large negative value, we should increase the likelihood of selecting that action.

### Statistics and reproducibility

Simulations lasting 30 days were carried out 10^5^ times for biogas production sensitivity analysis. Simulations lasting 20 days were carried out 10^4^ times for short-term production control. Simulations lasting 20 days were carried out 10^3^ times for long-term production and inventory control. Probability distribution, mean absolute error, coefficient of variance, and dynamic time warping loss were employed for statistical analyses of the results.

### Supplementary information

Supplementary Information

Description of Additional Supplementary Files

Supplementary Data

## Data Availability

The feedstock data supporting the findings of this study are available within the supplementary Data file.

## Code availability

The code for the reinforcement learning algorithms used in this work is available on GitHub at https://github.com/TongSustainabilityGroup/ADRL.
