# Supplementary material for: Reinforcement learning-based control for waste biorefining processes under uncertainty
Source: Commun Eng. 2024 Feb 29;3:38. doi: 10.1038/s44172-024-00183-7 (PMC10955880; doi:10.1038/s44172-024-00183-7)
Supplement: Supplementary file 1 — Supplementary Information [file 44172_2024_183_MOESM1_ESM.pdf]

Reinforcement Learning-Based Control for Waste Biorefining Processes Under Uncertainty

Ji Gao[1†], Abigael Wahlen[1†], Caleb Ju[2], Yongsheng Chen[3], Guanghui Lan[2*] and Zhaohui Tong[1*]

[1*]School of Chemical & Biomolecular Engineering, Georgia Institute of Technology, 311 Ferst Dr, Atlanta, 30318, GA, USA.
[2*]H. Milton Stewart School of Industrial and Systems Engineering, Georgia Institute of Technology, 755 Ferst Drive, Atlanta, 30332, GA, USA.
[3]School of Civil and Environmental Engineering, Georgia Institute of Technology, 790 Atlantic Dr, Atlanta, 30332, GA, USA.

*Corresponding author(s). E-mail(s): george.lan@isye.gatech.edu; zhaohui.tong@chbe.gatech.edu;
Contributing authors: jgao325@gatech.edu; awahlen3@gatech.edu; calebju4@gatech.edu; yongsheng.chen@ce.gatech.edu;
†These authors contributed equally to this work.

Supplementary Notes

Supplementary Note 1: Dynamic time warping loss

When comparing the degree of lagging between two time series in our problem, two facts should be considered. First, the two time series have the same length, and second, the alignment of the two time series should be monotonically increasing. The usual edit distances between sequences, such as Levenshtein distance, are not useful, hence, we apply the algorithm of dynamic time warping (DTW) [1, 2]. Dynamic time warping is an algorithm to create the best alignment between two sequences. Consider two sequences of the same length, the reference sequence $\{S^*\}_{t=1}^T$, and the sample sequence $\{S'\}_{t=1}^T$, the path of optimal alignment between the two sequences can be defined as:

$$A_{S',S^*} = (x_i, y_i)_{i=1}^M \in \arg\min_{(x_i,y_i)} \sum_{i=1}^M \|S^*_{x_i} - S'_{y_i}\|_2$$

$$s.t.\ (x_i, y_i)_{i=1}^M \text{ is warping path on a rank T square matrix}$$

The warping path $(x_i, y_i)_{i=1}^M$ satisfies three conditions, 1) boundary, $(x_1, y_1) = (1, 1), (x_M, y_M) = (T, T)$, 2) monotonicity, $x_i \leq x_{i+1}, y_i \leq y_{i+1}$, and 3) continuity, $(x_{i+1}, y_{i+1}) - (x_i, y_i) \in \{(1, 0), (0, 1), (1, 1)\}$. The dynamic time warping loss is defined as $\sum_{i=1}^M \|S^*_{x_i} - S'_{y_i}\|_2$ corresponds to the optimal alignment A_{S',S^*}. We used the python package FastDTW [3] for this calculation.

Supplementary Note 2: Major reaction pathways of anaerobic digestion

AD is a process where micro-organisms degrade organic materials in an oxygen-free environment and result in the production of biogas which in turn can be used to create fuel, energy, power, and electricity. Despite the wide adoption of this technology, its reaction mechanism is yet fully understood due to the complex reactions resulting in the creation of an array of inter-related intermediate products [4]. In order to generate biogas, the waste feedstock has to complete four main stages in the AD reaction, namely hydrolysis, acidogenesis, acetogenesis, and methanogenesis. Furthermore, differences in organic compounds (lipids, carbohydrates, and proteins) within the feedstock follow different reaction pathways. The intermediate products of each reaction step become the reactant of the next step, slowly leading to the degradation of complex organic molecules to simpler compounds like hydrogen and methane. In addition, different stages of reaction have different optimal conditions like pH and temperature, which further increases the complexity of each mechanism. The complexities within the reaction mechanisms boil down to uncertainties in biogas production, hence, effectively controlling the process for stable and resilient production becomes one of the main issues in the AD process.

Supplementary Note 3: Kinetics and modeling of anaerobic digestion

The anaerobic digestion model number 1 (ADM1) is the most widely accepted and most extensive model for AD [5]. Besides process dynamics, this model account of many factors that affect the process including pH, temperature, and chemical inhibitors. ADM1 described anaerobic digestion with five main steps, 1) disintegration, the initial concentration of the feedstock is broken down into three distinct substrates: lipids, proteins, and carbohydrates, 2) hydrolysis, these larger substrates are broken down into smaller pieces: lipids to long chain fatty acids, proteins to amino acids, and carbohydrates to simple sugars, 3) acidogenesis, the smaller biochemicals are broken down into acidic intermediates: acetate, butyrate, propionate, and valerate, 4) acetogenisis, the intermediate acids are formed into acetate and hydrogen, and 5) methanogenesis, conversion of acetate and hydrogen into methane and carbon dioxide. The kinetics model for this analysis is based on the ADM1 including soluble and insoluble components, inhibition, and liquid-gas transfer. It should be noted that the set of parameters needs to be further tuned for a specific process to adapt to its actual kinetics.

AD is a complex process influenced by numerous factors. We decided to use a widely used mechanistic model, ADM1, as the simulator for our study on RL-based control under feedstock uncertainty. This decision can be broken down into three justifications. 1) ADM1 has been used for various AD processes in the literature for fitting experimental data in sludge, wastewater, and co-digestion processes [6–9], which proves the applicability of the model in different AD processes. The Markov process in the RL framework is directly simulated using the ADM1 mechanistic model. 2) From a modeling perspective, for any factors that could significantly impact the production, modification of the model should be considered [10]. Otherwise, a proper parameter estimation of the ADM1 model can partially help with the intricacies. For example, microbial activity can be accounted for by adjusting the hydrolysis rate constants. Using the historical data from a local plant for parameter estimation can resemble the general characteristics of local waste for the inhibitions from microbial, metal ions, and other chemicals. More data from the real production process can enhance the robustness in dealing with more complex conditions. 3) We admit the fact that practical AD can encounter even more intricacies and challenges in modeling, spanning different biological, chemical, physical, and environmental factors, e.g., microbial growth dynamics, ions, density, particle size, mixing, foaming, etc. When creating a new RL algorithm for feedstock control, it is convenient to only consider a simplified system since we are focusing on the application RL in dealing with the uncertainties in feed heterogeneity. When a more comprehensive model is available, our RL algorithm can be applied upon training on local data.

Supplementary Note 4: Simulation Settings

We assumed a reactor with a 3400 cubic meters liquid holdup and 300 cubic meters overhead space. By assigning local controllers for the cooling, we assume the temperature of the reactor is kept around the favorable value of the AD process at 35°C, which left us the three incoming waste streams to be controlled in order to achieve the defined targets of methane production rate.

In the short-term scenario, the total flowrate of waste streams feeding into the reactor is limited between 120 and 390 cubic meters per day. The actions are discretized with an interval of 30 cubic meters, resulting in 1000 unique actions combining three feed sources. We combine two consecutive observations of the methane production rate as a single state in a moving horizon manner. This method will give a more accurate representation of the actual reactor condition by inherently incorporating gradient information at the cost of increased state space. The resulting size of discretized state space is 100, with single observation ranging from 40 to 300 cubic meters per day. An observation interval of 1 day is used.

For the long-term scenario with combined inventory control, the same observation interval is used. The state of the Markov decision process (MDP) model will now describe the biogas production rate and the amount of feedstock in each storage tank. The actions are the flowrates from each storage tanks to the reactor.

To make synthetic data for simulation purposes reflecting the seasonal variation, we need the overall shapes and relative quantities of three waste categories: FW, MSW, and AW for different seasons. The general profiles/shapes of the feed seasonal variation for MSW and AW are extracted from regional studies [11, 12] respectively for simulation purpose. AW consists of crops, manure, and other components with a cumulative annual volume of approximately 180 million metric tons in the U.S [13]. MSW production in the U.S. amounts to roughly 292 million metric tons [14], with the organic fraction of municipal solid waste (OFMSW) constituting around 28% of this total [15]. Additionally, FW, though sometimes categorized within MSW, is considered separately here. We consider an additional stream of FW originating from establishments such as restaurants and supermarkets, assuming this stream to be 50% of the OFMSW. Hence, the relative quantities of FW, MSW, and AW can be estimated at a ratio of approximately 1:2:4. We combine this ratio with estimated seasonal variability of MSW, FW and AW to create a bases for seasonal simulation. The simulated variation follows the equation below with 10% uniform random noise:

$$\begin{aligned}
AW &= 30 \times |\sin(\frac{\pi t}{360})| + 170 \\
MSW &= 30 \times (|\sin(\frac{\pi t}{360}) + \sin(\frac{\pi t}{120})| + 80 \\
FW &= 9 \times (|\sin(\frac{\pi t}{360}) + \sin(\frac{\pi t}{120}))| + 54.
\end{aligned}$$

The methane production is discretized with intervals of 30 from 10 to 310 cubic meters per day. The three storage tanks are discretized with intervals of 200 from 0 to 1000 cubic meters. The combined methane production and methane storage result in 2376 unique states. The combination of three flowrates results in a total number 225 actions, with total flowrates ranging from 40 to 460.

The weightings for the objective function in are set to be 0.5, 1, 0.1, 0.01. The parameters for the policy mirror descent algorithms defined in Equation 4 are $\eta = 0.75,\ \tau = 0,\ \mu = 0.1$. For the temporal difference estimation of the state-action-value function, the discount factor is 0.85, and the learning rate is 0.75.

Supplementary Note 5: Estimation of reference elemental data for proteins, lipids, carbohydrates, and lignins

There are different types and sizes of major biochemicals. While carbohydrates are mainly composed of similar base sugars, various types of lipids and proteins are commonly found in nature. To conduct our analysis, we specifically selected the most abundant types of lipids and proteins in plant and animal products. The proteins we chose for our analysis are gelatin, collagen, keratin, actin, tubulin, and hemoglobin, while our selection of lipids comprises GTO diglyceride, triglyceride, and steroid. We established enough standard compounds to determine the average values for carbon (C), oxygen (O), nitrogen (N), and hydrogen (H) in proteins (p), lipids (l), carbohydrates (c), and lignin (lig). Subsequently, we utilized these average values to fill in any missing elemental data. The formulas provided below were used to carry out our calculations.

$$\mathrm{C} = X_p f_{(\mathrm{C},p)} + X_c f_{(\mathrm{C},c)} + X_l f_{(\mathrm{C},l)} + X_{lig} f_{(\mathrm{C},lig)}$$
$$\mathrm{H} = X_p f_{(\mathrm{H},p)} + X_c f_{(\mathrm{H},c)} + X_l f_{(\mathrm{H},l)} + X_{lig} f_{(\mathrm{H},lig)}$$
$$\mathrm{O} = X_p f_{(\mathrm{O},p)} + X_c f_{(\mathrm{O},c)} + X_l f_{(\mathrm{O},l)} + X_{lig} f_{(\mathrm{O},lig)}$$
$$\mathrm{N} = X_p f_{(\mathrm{N},p)}$$

Where X is the given percentage of each biochemical type (proteins, lipids, carbohydrate, and lignin if applicable) and f describes the fraction of each element (C,H,O,N) in each biochemical. The average values calculated are proteins: %C=53.22, %H=6.29, %O=25.31, lipids: %C=69.4, %H=7.78, %O=17.35 , and Carbohydrates: %C=44.4, %H=6.17, %O=53.3. This analysis only accounts for the three main types of biochemicals found in nature. It does not account for contributions from the minor type of chemicals found in waste and nature. Furthermore, in the anaerobic digestive reactor, the main active components (and the main ones described by ADM1) are the distinctive biochemical components in this paper.

Supplementary Note 6: Sources for feedstock data

The feedstock data in Supplementary Data are from [16–56]

Supplementary Figures

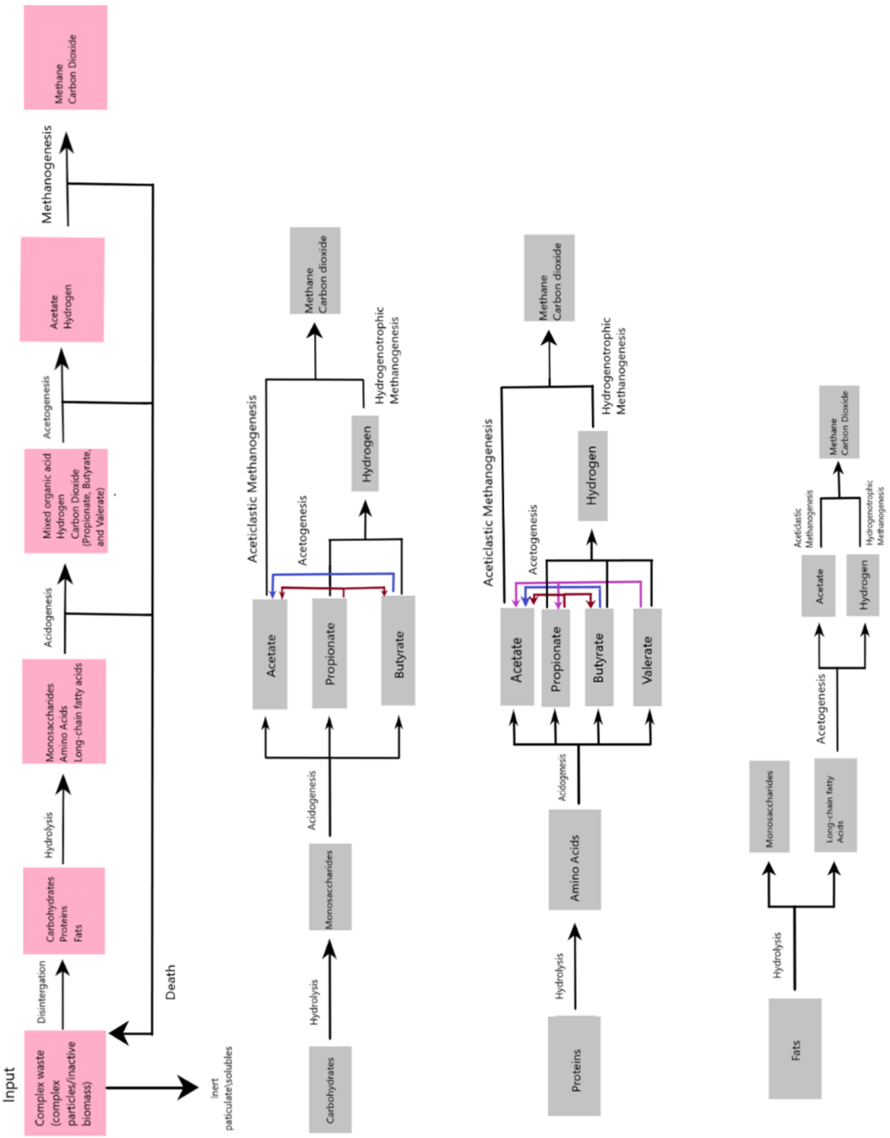

Supplementary Fig. 1 Major reaction pathways of anaerobic digestion for carbohydrates, proteins, and lipids

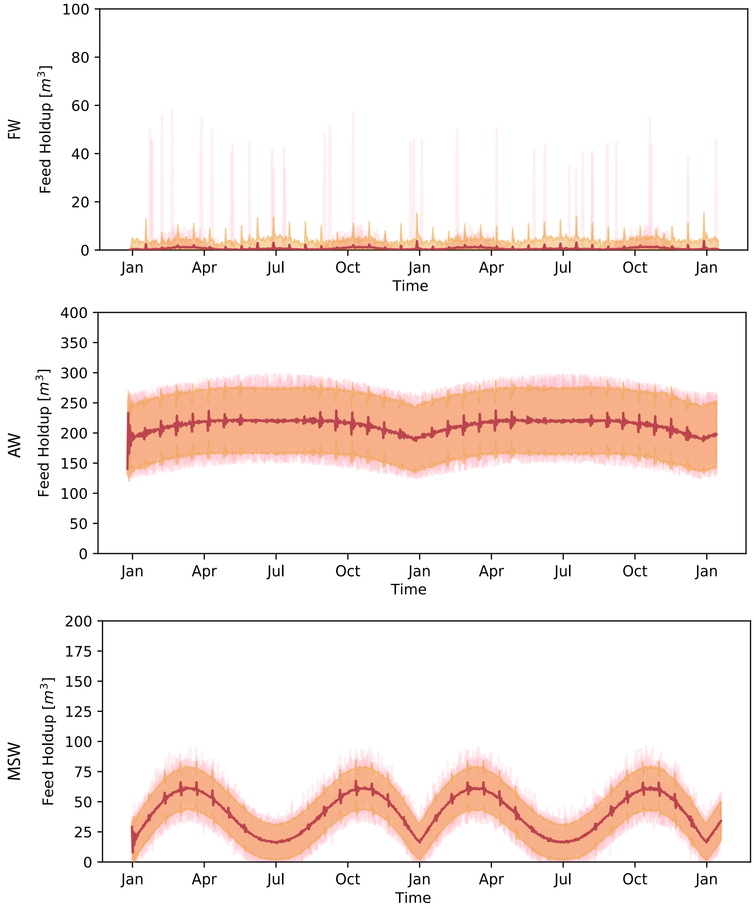

Supplementary Fig. 2 Storage holdups for FW, AW, and MSW for combined inventory and feed control via PMD algorithm. Red line: mean of the simulation results, orange shaded area: one standard deviation from the mean, pink lines: 10 sample trajectories of simulation results.

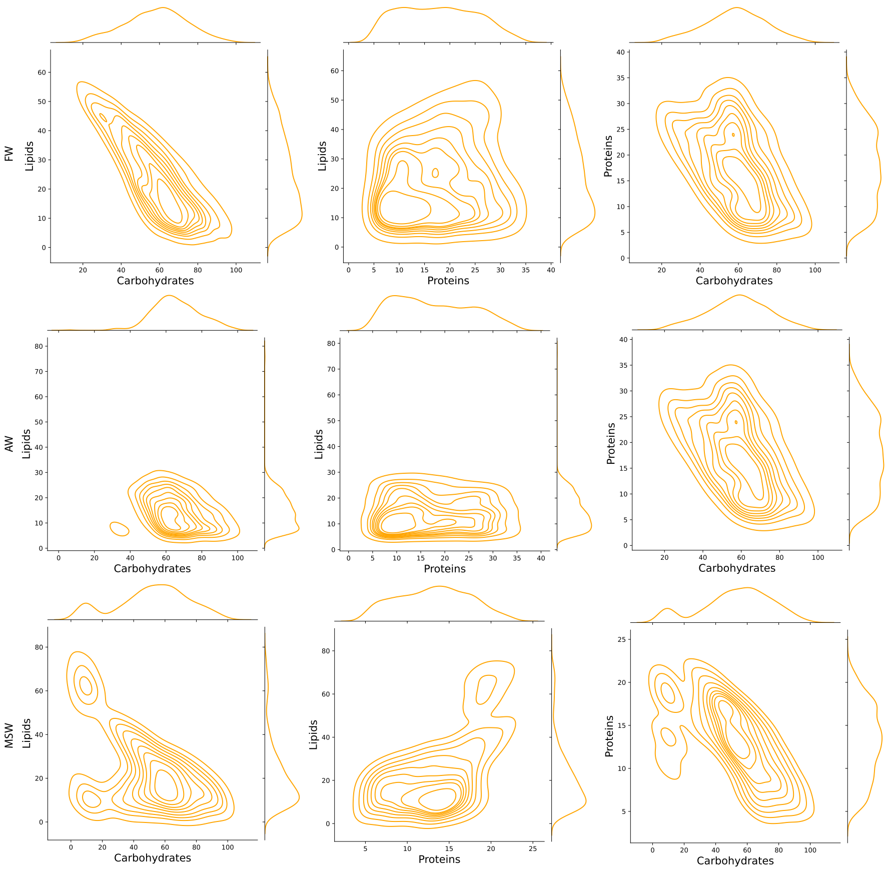

Supplementary Fig. 3 Projected distribution of biochemicals in FW, AW, and MSW. Contour lines within the box: 2D density distribution, Curves outside box: 1D density distribution.

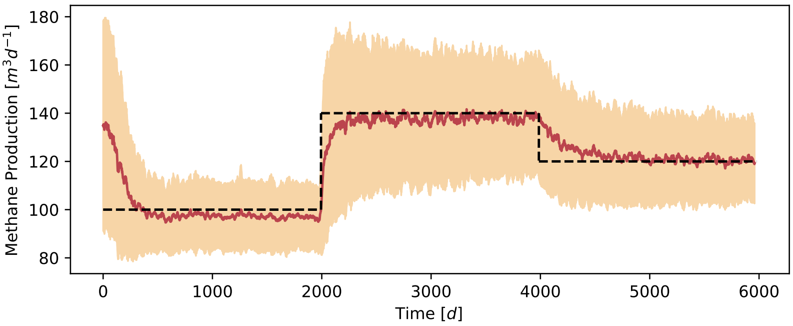

Supplementary Fig. 4 Demonstration of target changing controlled by the PMD algorithm Red line: mean of the simulation results, orange shaded area: one standard deviation from the mean, pink lines: 100 sample trajectories of simulation results.

Supplementary References

[1] T.K. Vintsyuk, Speech discrimination by dynamic programming. Cybernetics **4**(1), 52–57 (1968). https://doi.org/10.1007/BF01074755. URL https://doi.org/10.1007/BF01074755

[2] in *Information Retrieval for Music and Motion*, ed. by M. Müller (Springer, Berlin, Heidelberg, 2007), pp. 69–84. https://doi.org/10.1007/978-3-540-74048-3_4. URL https://doi.org/10.1007/978-3-540-74048-3_4

[3] tanitter. Slaypni/fastdtw (2019). URL https://github.com/slaypni/fastdtw

[4] F. Xu, Y. Li, X. Ge, L. Yang, Y. Li, Anaerobic digestion of food waste – Challenges and opportunities. Bioresource Technology **247**, 1047–1058 (2018). https://doi.org/10.1016/j.biortech.2017.09.020. URL https://www.sciencedirect.com/science/article/pii/S0960852417315687

[5] D.J. Batstone, I.W. Association (eds.), *Anaerobic Digestion Model No. 1: (ADM1)*, 1st edn. No. 13 in Scientific and Technical Report / IWA (IWA Publ, London, 2002)

[6] A. Catenacci, M. Grana, F. Malpei, E. Ficara, Optimizing ADM1 Calibration and Input Characterization for Effective Co-Digestion Modelling. Water **13**(21), 3100 (2021). https://doi.org/10.3390/w13213100. URL https://www.mdpi.com/2073-4441/13/21/3100

[7] K. Derbal, M. Bencheikh-lehocine, F. Cecchi, A.H. Meniai, P. Pavan, Application of the IWA ADM1 model to simulate anaerobic co-digestion of organic waste with waste activated sludge in mesophilic condition. Bioresource Technology **100**(4), 1539–1543 (2009). https://doi.org/10.1016/j.biortech.2008.07.064. URL https://linkinghub.elsevier.com/retrieve/pii/S0960852408006366

[8] D. Batstone, J. Keller, J. Steyer, A review of ADM1 extensions, applications, and analysis: 2002–2005. Water Science and Technology **54**(4), 1–10 (2006). https://doi.org/10.2166/wst.2006.520. URL https://iwaponline.com/wst/article/54/4/1/12796/A-review-of-ADM1-extensions-applications-and

[9] M.M. Otuzalti, N.A. Perendeci, Modeling of real scale waste activated sludge anaerobic digestion process by Anaerobic Digestion Model 1 (ADM1). International Journal of Green Energy **15**(7), 454–464 (2018). https://doi.org/10.1080/15435075.2018.1479265. URL https://www.tandfonline.com/doi/full/10.1080/15435075.2018.1479265

[10] R. Mo, W. Guo, D. Batstone, J. Makinia, Y. Li, Modifications to the anaerobic digestion model no. 1 (ADM1) for enhanced understanding and application of the anaerobic treatment processes – A comprehensive review. Water Research **244**, 120,504 (2023). https://doi.org/10.1016/j.watres.2023.120504. URL https://linkinghub.elsevier.com/retrieve/pii/S0043135423009442

[11] G. Denafas, T. Ruzgas, D. Martuzevičius, S. Shmarin, M. Hoffmann, V. Mykhaylenko, S. Ogorodnik, M. Romanov, E. Neguliaeva, A. Chusov, T. Turkadze, I. Bochoidze, C. Ludwig, Seasonal variation of municipal solid waste generation and composition in four East European cities. Resources, Conservation and Recycling **89**, 22–30 (2014). https://doi.org/10.1016/j.resconrec.2014.06.001. URL https://www.sciencedirect.com/science/article/pii/S092134491400130X

[12] J.F.M. Huijsmans, G.D. Vermeulen, J.M.G. Hol, P.W. Goedhart, A model for estimating seasonal trends of ammonia emission from cattle manure applied to grassland in the Netherlands. Atmospheric Environment **173**, 231–238 (2018). https://doi.org/10.1016/j.atmosenv.2017.10.050. URL https://www.sciencedirect.com/science/article/pii/S135223101730715X

[13] M. Downing, L.M. Eaton, R.L. Graham, M.H. Langholtz, R.D. Perlack, A.F. Turhollow Jr, B. Stokes, C.C. Brandt. U.s. billion-ton update: Biomass supply for a bioenergy and bioproducts industry. https://doi.org/10.2172/1023318. URL http://www.osti.gov/servlets/purl/1023318-ETXjs9/

[14] OLEM, EPA. National overview: Facts and figures on materials, wastes and recycling. URL https://www.epa.gov/facts-and-figures-about-materials-waste-and-recycling/national-overview-facts-and-figures-materials

[15] M. Sardarmehni, J.W. Levis, M.A. Barlaz, What is the best end use for compost derived from the organic fraction of municipal solid waste? **55**(1), 73–81. https://doi.org/10.1021/acs.est.0c04997. URL https://doi.org/10.1021/acs.est.0c04997. Publisher: American Chemical Society

[16] S. Suhartini, I. Nurika, R. Paul, L. Melville, Estimation of Biogas Production and the Emission Savings from Anaerobic Digestion of Fruit-based Agro-industrial Waste and Agricultural crops residues. BioEnergy Research **14**(3), 844–859 (2021). https://doi.org/10.1007/s12155-020-10209-5. URL https://link.springer.com/10.1007/s12155-020-10209-5

[17] L. Li, Q. He, X. Zhao, D. Wu, X. Wang, X. Peng, Anaerobic digestion of food waste: Correlation of kinetic parameters with operational

conditions and process performance. Biochemical Engineering Journal **130**, 1–9 (2018). https://doi.org/10.1016/j.bej.2017.11.003. URL https://linkinghub.elsevier.com/retrieve/pii/S1369703X17303108

[18] A. Galí, T. Benabdallah, S. Astals, J. Mata-Alvarez, Modified version of ADM1 model for agro-waste application. Bioresource Technology **100**(11), 2783–2790 (2009). https://doi.org/10.1016/j.biortech.2008.12.052. URL https://linkinghub.elsevier.com/retrieve/pii/S0960852408011346

[19] J.H. Ebner, R.A. Labatut, J.S. Lodge, A.A. Williamson, T.A. Trabold, Anaerobic co-digestion of commercial food waste and dairy manure: Characterizing biochemical parameters and synergistic effects. Waste Management **52**, 286–294 (2016). https://doi.org/10.1016/j.wasman.2016.03.046. URL https://linkinghub.elsevier.com/retrieve/pii/S0956053X16301301

[20] C. Zhao, H. Yan, Y. Liu, Y. Huang, R. Zhang, C. Chen, G. Liu, Bio-energy conversion performance, biodegradability, and kinetic analysis of different fruit residues during discontinuous anaerobic digestion. Waste Management **52**, 295–301 (2016). https://doi.org/10.1016/j.wasman.2016.03.028. URL https://linkinghub.elsevier.com/retrieve/pii/S0956053X1630112X

[21] Z. Zuo, S. Wu, W. Zhang, R. Dong, Performance of two-stage vegetable waste anaerobic digestion depending on varying recirculation rates. Bioresource Technology **162**, 266–272 (2014). https://doi.org/10.1016/j.biortech.2014.03.156. URL https://linkinghub.elsevier.com/retrieve/pii/S0960852414004611

[22] J.K. Cho, S.C. Park, H.N. Chang, Biochemical methane potential and solid state anaerobic digestion of Korean food wastes. Bioresource Technology **52**(3), 245–253 (1995). https://doi.org/10.1016/0960-8524(95)00031-9. URL https://linkinghub.elsevier.com/retrieve/pii/0960852495000319

[23] F. Cheng, M. Dehghanizadeh, M.A. Audu, J.M. Jarvis, F.O. Holguin, C.E. Brewer, Characterization and evaluation of guayule processing residues as potential feedstock for biofuel and chemical production. Industrial Crops and Products **150**, 112,311 (2020). https://doi.org/10.1016/j.indcrop.2020.112311. URL https://linkinghub.elsevier.com/retrieve/pii/S0926669020302272

[24] F. Codignole Luz, M. Volpe, L. Fiori, A. Manni, S. Cordiner, V. Mulone, V. Rocco, Spent coffee enhanced biomethane potential via an integrated hydrothermal carbonization-anaerobic digestion process. Bioresource Technology **256**, 102–109 (2018). https://doi.org/10.1016/j.biortech.2018.02.021. URL https://linkinghub.elsevier.com/retrieve/pii/S0960852418301950

[25] A. Kumar, A. Ramanathan, Theoretical analysis involved in the prediction of biomethane production from fruit wastes through anaerobic digestion. Materials Today: Proceedings **46**, 9788–9793 (2021). https://doi.org/10.1016/j.matpr.2020.10.779. URL https://linkinghub.elsevier.com/retrieve/pii/S2214785320384029

[26] M. Carmona-Cabello, I.L. Garcia, D. Leiva-Candia, M.P. Dorado, Valorization of food waste based on its composition through the concept of biorefinery. Current Opinion in Green and Sustainable Chemistry **14**, 67–79 (2018). https://doi.org/10.1016/j.cogsc.2018.06.011. URL https://linkinghub.elsevier.com/retrieve/pii/S2452223618300361

[27] M. Carmona-Cabello, I. García, J. Sáez-Bastante, S. Pinzi, A. Koutinas, M. Dorado, Food waste from restaurant sector – Characterization for biorefinery approach. Bioresource Technology **301**, 122,779 (2020). https://doi.org/10.1016/j.biortech.2020.122779. URL https://linkinghub.elsevier.com/retrieve/pii/S0960852420300481

[28] R. Posmanik, D. Cantero, A. Malkani, D. Sills, J. Tester, Biomass conversion to bio-oil using sub-critical water: Study of model compounds for food processing waste. The Journal of Supercritical Fluids **119**, 26–35 (2017). https://doi.org/10.1016/j.supflu.2016.09.004. URL https://linkinghub.elsevier.com/retrieve/pii/S0896844616302923

[29] R. Chandra, H. Takeuchi, T. Hasegawa, Methane production from lignocellulosic agricultural crop wastes: A review in context to second generation of biofuel production. Renewable and Sustainable Energy Reviews **16**(3), 1462–1476 (2012). https://doi.org/10.1016/j.rser.2011.11.035. URL https://linkinghub.elsevier.com/retrieve/pii/S1364032111005818

[30] R. Girault, G. Bridoux, F. Nauleau, C. Poullain, J. Buffet, J.P. Steyer, A. Sadowski, F. Béline, A waste characterisation procedure for ADM1 implementation based on degradation kinetics. Water Research **46**(13), 4099–4110 (2012). https://doi.org/10.1016/j.watres.2012.04.028. URL https://linkinghub.elsevier.com/retrieve/pii/S0043135412002837

[31] K. Tsigkou, D. Zagklis, P. Tsafrakidou, P. Zapanti, G. Manthos, K. Karamitou, C. Zafiri, M. Kornaros, Expired food products and used disposable adult nappies mesophilic anaerobic co-digestion: Biochemical methane potential, feedstock pretreatment and two-stage system performance. Renewable Energy **168**, 309–318 (2021). https://doi.org/10.1016/j.renene.2020.12.062. URL https://www.sciencedirect.com/science/article/pii/S0960148120320000

[32] P. Moretti, J. Morais de Araujo, A. Borges de Castilhos, P. Buffière, R. Gourdon, R. Bayard, Characterization of municipal biowaste categories for their capacity to be converted into a feedstock aqueous slurry

to produce methane by anaerobic digestion. Science of The Total Environment **716**, 137,084 (2020). https://doi.org/10.1016/j.scitotenv.2020.137084. URL https://www.sciencedirect.com/science/article/pii/S0048969720305945

[33] K.R. Parmar, A.B. Ross, Integration of Hydrothermal Carbonisation with Anaerobic Digestion; Opportunities for Valorisation of Digestate. Energies **12**(9), 1586 (2019). https://doi.org/10.3390/en12091586. URL https://www.mdpi.com/1996-1073/12/9/1586

[34] J. Neumann, J. Meyer, M. Ouadi, A. Apfelbacher, S. Binder, A. Hornung, The conversion of anaerobic digestion waste into biofuels via a novel Thermo-Catalytic Reforming process. Waste Management **47**, 141–148 (2016). https://doi.org/10.1016/j.wasman.2015.07.001. URL https://linkinghub.elsevier.com/retrieve/pii/S0956053X15300180

[35] H. Li, D. Mehmood, E. Thorin, Z. Yu, Biomethane Production Via Anaerobic Digestion and Biomass Gasification. Energy Procedia **105**, 1172–1177 (2017). https://doi.org/10.1016/j.egypro.2017.03.490. URL https://linkinghub.elsevier.com/retrieve/pii/S1876610217305313

[36] Y. Zhang, C.J. Banks, S. Heaven, Anaerobic digestion of two biodegradable municipal waste streams. Journal of Environmental Management **104**, 166–174 (2012). https://doi.org/10.1016/j.jenvman.2012.03.043. URL https://linkinghub.elsevier.com/retrieve/pii/S0301479712001624

[37] Y. Yang, S. Heaven, N. Venetsaneas, C. Banks, A. Bridgwater, Slow pyrolysis of organic fraction of municipal solid waste (OFMSW): Characterisation of products and screening of the aqueous liquid product for anaerobic digestion. Applied Energy **213**, 158–168 (2018). https://doi.org/10.1016/j.apenergy.2018.01.018. URL https://linkinghub.elsevier.com/retrieve/pii/S0306261918300151

[38] F. Monlau, M. Francavilla, C. Sambusiti, N. Antoniou, A. Solhy, A. Libutti, A. Zabaniotou, A. Barakat, M. Monteleone, Toward a functional integration of anaerobic digestion and pyrolysis for a sustainable resource management. Comparison between solid-digestate and its derived pyrochar as soil amendment. Applied Energy **169**, 652–662 (2016). https://doi.org/10.1016/j.apenergy.2016.02.084. URL https://linkinghub.elsevier.com/retrieve/pii/S0306261916302240

[39] Z.J. Yong, M.J. Bashir, M.S. Hassan, Assessment of environmental, energy and economic prospective of anaerobic digestion of organic municipal solid waste in Malaysia. IOP Conference Series: Earth and Environmental Science **463**(1), 012,054 (2020). https://doi.org/10.1088/1755-1315/463/1/012054. URL https://iopscience.iop.org/article/10.1088/1755-1315/463/1/012054

[40] M. Mastellone, in *17th International Conference on Waste Management and Landfill SymposiumAt: Forte Village Sardegna* (2019)

[41] R. Bayard, H. Benbelkacem, R. Gourdon, P. Buffière, Characterization of selected municipal solid waste components to estimate their biodegradability. Journal of Environmental Management **216**, 4–12 (2018). https://doi.org/10.1016/j.jenvman.2017.04.087. URL https://linkinghub.elsevier.com/retrieve/pii/S0301479717304425

[42] A.E. Brown, J.S. Ford, C.S. Bale, M.A. Camargo-Valero, N.J. Cheffins, P.E. Mason, A.M. Price-Allison, A.B. Ross, P.G. Taylor, An assessment of road-verge grass as a feedstock for farm-fed anaerobic digestion plants. Biomass and Bioenergy **138**, 105,570 (2020). https://doi.org/10.1016/j.biombioe.2020.105570. URL https://linkinghub.elsevier.com/retrieve/pii/S0961953420301045

[43] C. Teater, Z. Yue, J. MacLellan, Y. Liu, W. Liao, Assessing solid digestate from anaerobic digestion as feedstock for ethanol production. Bioresource Technology **102**(2), 1856–1862 (2011). https://doi.org/10.1016/j.biortech.2010.09.099. URL https://linkinghub.elsevier.com/retrieve/pii/S0960852410016317

[44] P. Biernacki, S. Steinigeweg, A. Borchert, F. Uhlenhut, Application of Anaerobic Digestion Model No. 1 for describing anaerobic digestion of grass, maize, green weed silage, and industrial glycerine. Bioresource Technology **127**, 188–194 (2013). https://doi.org/10.1016/j.biortech.2012.09.128. URL https://linkinghub.elsevier.com/retrieve/pii/S0960852412014903

[45] D. Jerger, D. Chynoweth, H. Isaacson, Anaerobic digestion of sorghum biomass. Biomass **14**(2), 99–113 (1987). https://doi.org/10.1016/0144-4565(87)90013-8. URL https://linkinghub.elsevier.com/retrieve/pii/0144456587900138

[46] B. Jha, R. Chandra, V.K. Vijay, P.M. Subbarao, A. Isha, Utilization of de-oiled rice bran as a feedstock for renewable biomethane production. Biomass and Bioenergy **140**, 105,674 (2020). https://doi.org/10.1016/j.biombioe.2020.105674. URL https://linkinghub.elsevier.com/retrieve/pii/S0961953420302087

[47] X. Chen, Y. Gu, X. Zhou, Y. Zhang, Asparagus stem as a new lignocellulosic biomass feedstock for anaerobic digestion: Increasing hydrolysis rate, methane production and biodegradability by alkaline pretreatment. Bioresource Technology **164**, 78–85 (2014). https://doi.org/10.1016/j.biortech.2014.04.070. URL https://linkinghub.elsevier.com/retrieve/pii/S0960852414006105

[48] R. Bedoić, L. Čuček, B. Ćosić, D. Krajnc, G. Smoljanić, Z. Kravanja, D. Ljubas, T. Pukšec, N. Duić, Green biomass to biogas – A study on anaerobic digestion of residue grass. Journal of Cleaner Production **213**, 700–709 (2019). https://doi.org/10.1016/j.jclepro.2018.12.224. URL https://linkinghub.elsevier.com/retrieve/pii/S095965261833943X

[49] P.S. Calabrò, R. Greco, A. Evangelou, D. Komilis, Anaerobic digestion of tomato processing waste: Effect of alkaline pretreatment. Journal of Environmental Management **163**, 49–52 (2015). https://doi.org/10.1016/j.jenvman.2015.07.061. URL https://linkinghub.elsevier.com/retrieve/pii/S0301479715302048

[50] J. Lee, K.Y. Park, J. Cho, E.E. Kwon, J.Y. Kim, Anaerobic digestion as an alternative disposal for phytoremediated biomass from heavy metal contaminated sites. Environmental Pollution **243**, 1704–1709 (2018). https://doi.org/10.1016/j.envpol.2018.09.108. URL https://linkinghub.elsevier.com/retrieve/pii/S0269749118316208

[51] Steffen, (2000). URL https://www.semanticscholar.org/paper/Feedstocks-for-Anaerobic-Digestion-Steffen/293ea2a9a8d8315548b8d749f60e4b28378d9dfa

[52] Phyllis2 - Database for the physico-chemical composition of (treated) lignocellulosic biomass, micro- and macroalgae, various feedstocks for biogas production and biochar. URL https://phyllis.nl/

[53] S. Achinas, G.J.W. Euverink, Theoretical analysis of biogas potential prediction from agricultural waste. Resource-Efficient Technologies **2**(3), 143–147 (2016). https://doi.org/10.1016/j.reffit.2016.08.001. URL http://linkinghub.elsevier.com/retrieve/pii/S2405653716300264

[54] A.N. Matheri, F. Ntuli, J.C. Ngila, T. Seodigeng, C. Zvinowanda, C.K. Njenga, Quantitative characterization of carbonaceous and lignocellulosic biomass for anaerobic digestion. Renewable and Sustainable Energy Reviews **92**, 9–16 (2018). https://doi.org/10.1016/j.rser.2018.04.070. URL https://linkinghub.elsevier.com/retrieve/pii/S1364032118302806

[55] J.J.E.. Rea, Kinetic modeling and experimentation of anaerobic digestion. Thesis, Massachusetts Institute of Technology (2014). URL https://dspace.mit.edu/handle/1721.1/92070

[56] N. Wang, D. Huang, C. Zhang, M. Shao, Q. Chen, J. Liu, Z. Deng, Q. Xu, Long-term characterization and resource potential evaluation of the digestate from food waste anaerobic digestion plants. Science of The Total Environment **794**, 148,785 (2021). https://doi.org/10.1016/j.scitotenv.2021.148785. URL https://linkinghub.elsevier.com/retrieve/pii/S0048969721038572
