# Supplementary material for: Reinforcement learning-based control for waste biorefining processes under uncertainty
Source: Commun Eng. 2024 Feb 29;3:38. doi: 10.1038/s44172-024-00183-7 (PMC10955880; doi:10.1038/s44172-024-00183-7)
Supplement: Supplementary file 2 — Description of Additional Supplementary Files [file 44172_2024_183_MOESM2_ESM.pdf]

Description of Additional Supplementary Files

File name: Supplementary Data 1

Description: The feedstock data supporting the findings of this study
